# Foliar Litter Nitrogen Dynamics as Affected by Forest Gap in the Alpine Forest of Eastern Tibet Plateau

**DOI:** 10.1371/journal.pone.0097112

**Published:** 2014-05-12

**Authors:** Qiqian Wu, Fuzhong Wu, Wanqin Yang, Yeyi Zhao, Wei He, Bo Tan

**Affiliations:** 1 Institute of Ecological Forestry, Sichuan Agricultural University, Chengdu, Sichuan, China; 2 Center for Ecological Research, Northeast Forestry University, Harbin, Heilongjiang, China; DOE Pacific Northwest National Laboratory, United States of America

## Abstract

There is increasing attention on the effects of seasonal snowpack on wintertime litter decomposition, as well as the processes following it, in cold biomes. However, little information is available on how litter nitrogen (N) dynamics vary with snowpack variations created by tree crown canopies in alpine forests. Therefore, to understand the effects of seasonal snowpack on litter N dynamics during different critical stages, litterbags with fir (*Abies faxoniana*), birch (*Betula albo-sinensis*), larch (*Larix mastersiana*) and cypress (*Sabina saltuaria*) foliar litter were placed on the forest floor beneath snowpack created by forest gaps in the eastern Tibet Plateau. The litterbags were sampled at the onset of freezing, deep freezing, thawing and growing stages from October 2010 to October 2012. Mass loss and N concentrations in litter were measured. Over two years of decomposition, N release occurred mainly during the first year, especially during the first winter. Litter N release rates (both in the first year and during the entire two-year decomposition study period) were higher in the center of canopy gaps than under closed canopy, regardless of species. Litter N release rates in winter were also highest in the center of canopy gaps and lowest under closed canopy, regardless of species, however the reverse was found during the growing season. Compared with broadleaf litter, needle litter N release comparisons of gap center to closed canopy showed much stronger responses to the changes in snow cover in winter and availability of sunshine during the growing season. As the decomposition proceeded, decomposing litter quality, microbial biomass and environmental temperature were important factors related to litter N release rate. This suggests that if winter warm with climate change, reduced snow cover in winter might slow down litter N release in alpine forest.

## Introduction

Nitrogen (N) released from plant litter plays a crucial role in maintaining soil fertility and ecosystem productivity in most terrestrial ecosystems [1], especially in N-limited ecosystems [2], [3]. The present consensus is that vegetation type (referred to as litter quality) is the primary determining factor in the rate of N release during decomposition at the local scale, and climate (temperature, moisture, vegetation and soil types) controls the global-scale patterns in N release during decomposition [1], [4], [5]. However, increasingly well-documented evidence has accumulated demonstrating that seasonal snowpack and the related seasonal freeze-thaw pattern have strong effects on wintertime litter decomposition, microbial activity, and the following decomposition process in cold biomes [6]–[10]. According to IPCC [11], ongoing winter warming and extreme weather events are changing the patterns of seasonal snowpack and freeze-thaw cycles in cold biomes, and subsequently affected matter cycling. Therefore recent research has directed much more attention to the effects of simulated changes in seasonal snowpack and freeze-thaw cycles on litter decomposition and soil biological and biochemical processes [8]–[10], [12]. At the local scale, however, the dynamic pattern of seasonal snowpack in the high-altitude frigid forest ecosystem is greatly influenced by forest gaps and crown canopies. However, so far not a lot of information has been available on the affects of seasonal snowpack gradient created by forest gaps and crown in winter canopies on nitrogen dynamics.

In high-altitude frigid forest ecosystems, the interception of canopy on snow accumulation and the effects of canopy shading on snow ablation in winter often create a snowpack gradient on the forest floor between the center of a gap and under the forest canopy [13]. However, snowpack gradients vary during different critical stages. In deep winter, snowpack depth decreases from the gap center to under the crown canopy, owing to the crown canopy intercepting snow and low temperatures; while in late winter and early spring, snowpack depth increases from the gap center to under the canopy, owing to the shading sunlight that the crown canopy provides, which leads to a lower rate of snow ablation beneath the canopy. The differences in the dynamic pattern of snow accumulation and ablation from the gap center to under the canopy are influenced by the pattern of seasonal freeze-thaw cycles during different stages of the year [5]. In the growing season the crown canopy intercepts rainfall and creates shade, which and subsequently effects transpiration, and therefore also affects the dynamics of soil moisture and temperature [14]. Some studies have shown that large gaps significantly reduce microbial activity and decomposition rates by changing environmental conditions that should consequently reduce N cycling rates in a subtropical forest ecosystem [15]. Theoretically, the effect of forest gaps on litter decay and N release during the snowy season is different from those in the growing season, owing to both the canopy intercepting snow and reducing its accumulation, and the snowpack providing insulation. In addition, the accumulation and ablation of snow and ice in forest gaps compared with under canopies may affect the process of N dynamics in litter through leaching, mechanical disruption, and the effects of biological activity. As yet, little information has been available on the effect of winter snowpack created by forest gaps and crown canopies on the dynamics of litter N release.

Litter quality is the internal determining factor regarding the rate of early litter decomposition, especially as it relates to N release from foliar litter [16], [17]. Foliar litter with lower C/N and lignin/N ratios are most favorable for microbial growth and invertebrate digestion. In turn, they have higher rates of decay and N release [17]. Theoretically, the process of foliar litter decay in cold biomes differs from warm biomes that are not influenced by seasonal snowpack snowmelt leaching, mechanical disruption of freeze-thaw cycles, and the influence of microbial degradation of cryophile and cold resistant microorganisms Recently, Zhu et al. [9] have demonstrated that frequent freeze-thaw cycles and litter chemical properties determine winter decomposition, while microbe-related factors play more important roles in determining decomposition during the subsequent growing season. This implies that the effects of litter quality on litter N release in winter might depend on the pattern of seasonal snowpack and freeze-thaw cycles.

The alpine forest located in the upper reaches of Yangtze River and eastern Tibet Plateau plays important roles in holding headwater, conserving soil, nursing biodiversity, regulating regional climate, sequestering carbon dioxide and indicating climate change [18]. This alpine forest ecosystem is characterized by seasonal snowpack and freeze-thaw cycles [8], a thick soil organic layer and a thin mineral soil layer [19]. Forest regeneration occurs mainly in forest gaps, and therefore is also influenced by the corresponding snowpack gradient conditions created by forest gaps and crown canopies in winter [13]. Mass loss, nutrient release, and microbial biomass during the process of wintertime litter decomposition have been widely investigated in alpine forests [8]–[10], [20], [21]. Based on these previous investigations, it is hypothesized that N release rates from foliar litter will decrease along the snowpack gradient from the gap center to canopy-cover in winter, but the opposite will occur during the growing season.

To test this hypothesis, litterbags with fir (*Abies faxoniana*), birch (*Betula albo-sinensis*), larch (*Larix mastersiana*) and cypress (*Sabina saltuaria*) foliar litter were placed on the forest floor beneath different depths of winter snowpack created by forest gap and crown canopy in the Bipenggou Nature Reserve, Sichuan, China (which is located in the upper reaches of Yangtze River and eastern Tibet Plateau) on October 26, 2010. We aim to understand the litter nitrogen dynamics in response to forest gaps in the non-growing and growing seasons.

## Materials and Methods

### Ethics Statement

The Institute of Ecological Forestry, Sichuan Agricultural University, received a permit from the Western Sichuan Forestry Bureau to conduct scientific experiments in the Bipenggou Nature Reserve since March 2006. The senescent fresh foliar litter collected for this study were only sampled at a very limited scale, and thus had negligible effects on broader ecosystem functioning. Moreover, this research was carried out in compliance with the laws of the People's Republic of China. The research did not involve measurements of humans or animals and no endangered or protected plant species were involved.

### Site Description

This study site is located in the Bipenggou Nature Reserve (E102°53′–102°57′, N31°14′–31°19′, 2458–4619 m a.s.l.), a transitional area between the Qinghai-Tibet Plateau and the Sichuan Basin, southwest China [9]. The mean annual temperature ranges from 2°C to 4°C, with maximum and minimum temperatures of 23°C and −18°C. The annual precipitation is approximately 850 mm. The forest soil is classified as Cambisols and Primosols according to Gong et al. [22]. The snow cover season is from November to April of the following year. The main forest vegetation is *Abies faxoniana* primary forest. *A. faxoniana*, *Betula albo-sinensis*, *Larix mastersiana* and *Sabina saltuaria* are representative forest species. The understory plants are dominated by *Festuca ovin*, *Rhododendron delavayi*, *Carex* spp., *Cystopteris montana* and *Berberis sargentiana* [8], [9]. Shade density is about 0.7, and the average tree age is 130 a. In this site, extended gaps and canopy gaps accounted for a total forest landscape area of 12.60 and 23.05% [23].

### Foliar Litter Decomposition Experiment

In September 2010, fresh senescent leaves of fir, cypress, larch and birch were collected from the corresponding forest floors. To avoid damaging the structure of the litter during oven-drying, the fresh foliar litter was air-dried for more than 2 weeks at room temperature. Dry weight of litter was determined by oven-drying (65°C, 48 h). Samples of the air-dried foliar litter were placed inside nylon mesh bags (20×20 cm, with 0.055 mm mesh size placed on the forest floor, 1 mm mesh size placed on the surface, and 10 g per bag), and the edges were sealed [24], [25]. A total of 3000 litter bags (5 subplots ×4 species ×10 sampling date ×5 replicates ×3 gaps) were prepared. The initial characteristics of foliar litter of the four species are listed in the Table 1.

**Table 1:** Initial chemical characteristics of foliar litter of fir (*Abies faxoniana*), larch (*Larix mastersiana*), cypress (*Sabina saltuaria*), and birch (*Betula albo-sinensis*) in the alpine forest.

Species	Total C/(g·kg^−1^)	Total N/(g·kg^−1^)	Total P/(g·kg^−1^)	Lignin (L)/(%)	Cellulose/(%)	C/N	C/P	N/P	L/N
Fir	520.35 (4.35)a	11.20 (0.22)b	1.39 (0.07)b	25.06 (0.42)c	10.64 (0.41)b	46.46 (0.50)c	374.35 (14.54)b	8.06 (0.23)b	22.37 (0.06)c
Larch	515.71 (2.37)ab	8.98 (0.33)d	2.49 (0.06)a	32.39 (0.39)b	10.08 (0.53)b	57.43 (1.83)a	207.02 (3.67)d	3.60 (0.05)d	36.07 (0.87)a
Cypress	512.58 (1.38)ab	9.90 (0.41)c	1.48 (0.02)b	21.60 (0.41)d	10.85 (0.65)ab	51.78 (2.02)b	346.20 (4.76)c	6.68 (0.17)c	21.82 (0.50)c
Birch	514.80 (2.38)b	14.65 (0.23)a	1.04 (0.05)c	37.29 (0.53)a	11.73 (0.68)a	35.14 (0.39)d	494.73 (19.81)a	14.08 (0.41)a	25.45 (0.04b)

The same letter in the same column indicates no significant difference at 0.05 level.

*n* = 5.

**Figure 1:**
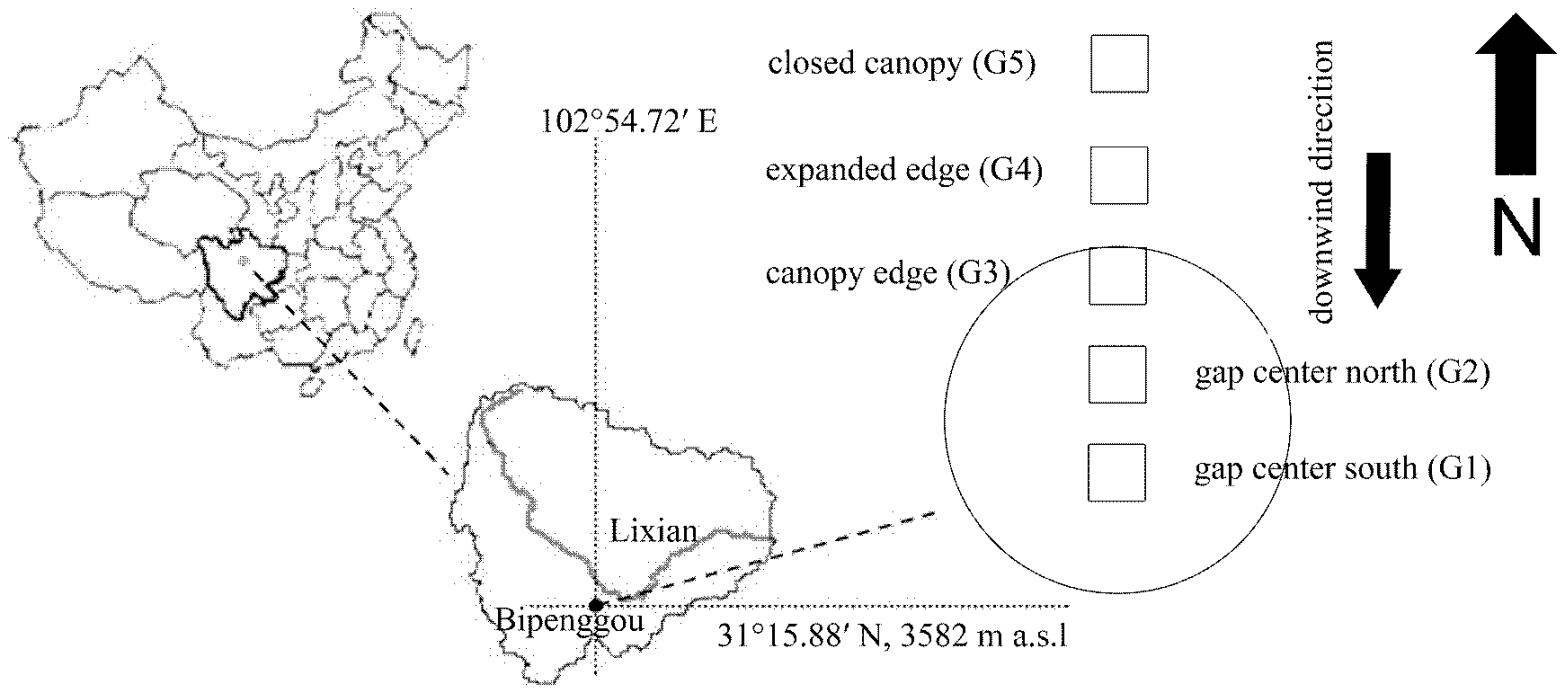
Experimental layout in all experimental gaps by gap position. G1 to G5 were located from gap center to closed canopy. Abbreviations: G1 = gap 1, gap center south; G2 = gap 2, gap center north; G3 = gap 3, canopy edge; G4 = gap 4, expanded edge; G5 = gap 5, closed canopy.

Field investigation and previous local data were used to randomly select three 25×25 m sampling plots in *Abies faxoniana* primary forests on October 26, 2010. The total area of these plots was 3 hm^2^ These plots constituted the experimental unit. Along the downwind direction, five subplots (4×4 m) were set at 3- to 4-m intervals from the south gap center to under the closed canopy in each plot. The symbols of G1 (gap 1, gap center south), G2 (gap 2, gap center north), G3 (gap 3, canopy edge), G4 (gap 4, expanded edge) and G5 (gap 5, closed canopy) signify the subplots reaching from the forest gap center to under the crown canopy (Figure 1). All litterbags with each of the four tree species were placed on the floor of each subplot (50 bags per subplot), with at least 2 cm intervals between each litterbag to avoid mutual disturbance.

To qualify the N released from foliar litter at different critical stages, we sampled litterbags 10 times over 2 years based on the field investigation and previous local data: December 23, 2010 (the 1^st^ onset of freezing stage, OF1); March 3, 2011 (the 1^st^ deep freezing stage, DF1); April 19, 2011 (the 1^st^ thawing stage, TS1); August 19, 2011 (the 1^st^ early growing season, EGS1); November 8, 2011 (the 1^st^ late growing season, LGS1); December 27, 2011 (the 2^nd^ onset freezing stage, OF2); March 7, 2012 (the 2^nd^ deep freezing stage, DF2); April 28, 2012 (the 2^nd^ thawing stage, TS2); August 25, 2012 (the 2^nd^ early growing season, EGS2); October 29, 2012 (the 2^nd^ late growing season, LGS2). Three litterbags per tree species were collected from each of the sampling subplots.

**Figure 2:**
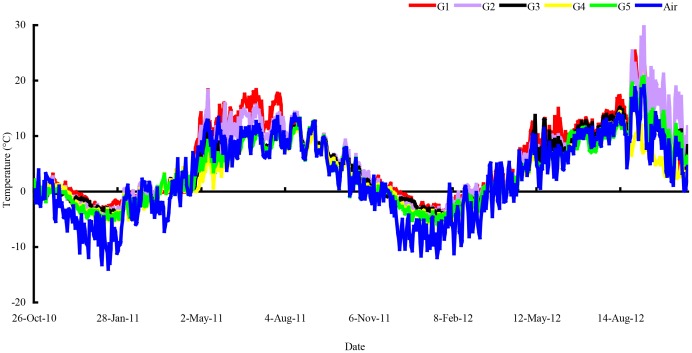
Dynamics of soil and air temperature during litter decomposition from 26 October 2010 to 29 October 2012. Abbreviations: G1 = gap 1, gap center south; G2 = gap 2, gap center north; G3 = gap 3, canopy edge; G4 = gap 4, expanded edge; G5 = gap 5, closed canopy.

**Figure 3:**
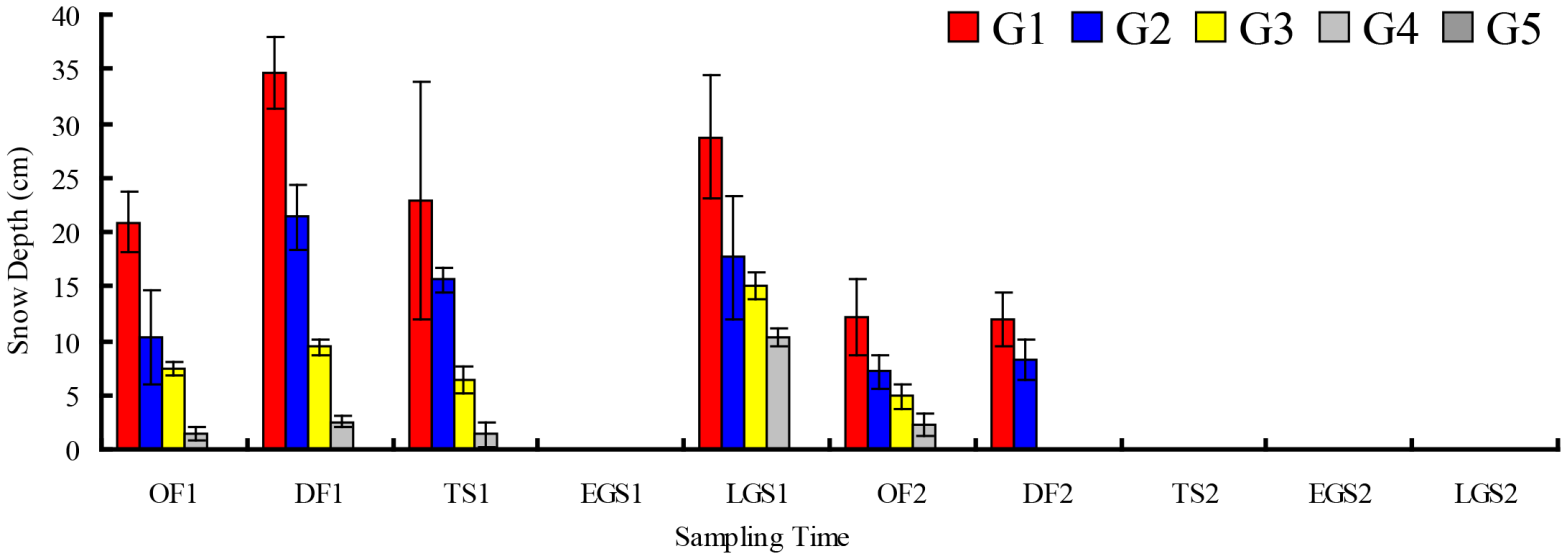
Depth changes of winter snowpack in different sampling time (mean ± *SD*, *n* = 5). Abbreviations: OF = onset of freezing stage; DF = deep freezing stage; TS = thawing stage; EGS = early growing season; LGS = late growing season; G1 = gap 1, gap center south; G2 = gap 2, gap center north; G3 = gap 3, canopy edge; G4 = gap 4, expanded edge; G5 = gap 5, closed canopy.

Temperature in the litterbags was measured every 2 h from October 26, 2010 to October 28, 2012 using an iButton DS1923-F5 Recorder (Maxim Integrated Products, Inc., Sunnyvale, TX, USA) (Figure 2). A freeze-thaw cycle was defined whenever the temperature dropped below 0°C for at least 3 h and followed by a rise above 0°C for at least 3 h, and vice versa [26]. In previous research, we have found that the day and night temperature fluctuated greatly. This means that, compared with the other temperature indices, accumulated positive and negative temperature could have a more profound effect on the soil ecological process [27], indicating that accumulated positive and negative temperature were related to seasonal snow cover. Accumulated temperature, and accumulated positive and negative temperatures in the surface soil layer were therefore calculated based on the daily mean temperature [28], and the relationship between litter quality, temperature indices, microbial biomass and N release rates were analyzed. Snowpack depth from gap center to closed canopy was measured at sampling litter bags in winter. Owing to the limits of experimental conditions and winter observations, snow depth was obtained by directly measuring with a ruler, and then the mean value was calculated (Figure 3).

### Chemical Analysis

Samples were stored at 4°C and analyses were finished within 2 weeks. Foreign materials such as the ingrown roots, soil debris and microbial hyphen in the litterbags were carefully removed. The sampled litter was oven-dried at 70°C for 48 h to a constant weight, and then ground (1 mm sieve) for N analysis [29].

### Microbial Biomass Analysis

Microbial biomass carbon (MBC) and microbial biomass N (MBN) in litter were determined according to the differences between unfumigated and fumigated samples by the dichromate oxidation–ferrous sulphate titration method and indigotic colorimetry method following extraction with 0.5 mol L^−1^ K_2_SO_4_, respectively. Efficiency factor (*Kc* = 0.38) was used to correct for the incomplete extractability. The correction factors were *K*_C_ = 0.30 for MBC and *K*_N_ = 0.45 for MBN, respectively [30], [31].

### Statistical Analysis

The net release rate of N:
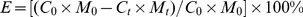
(1)Where: *E* was the net release rate of N (%), *C_t_* was the concentration of N at the time *t*, *M_t_* was the dry weight of litter at the time t (g), *C_0_* was the initial concentration of N (mg·g^−1^), *M_0_* was the initial litter weight in bags when they were placed on the floor of each subplot. When N was net release, *E* was a positive value, and reciprocally, when N was net enrichment, *E* was a negative value.

The relative proportion at each decomposition stage to the whole two-year N release was:
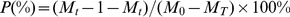
(2)Where: *M*_0_ was N mass of dry litter before the bags were placed on the floor of each subplot (g), (*M*_t-1_ *-M*_t_) was N mass of two adjoining time (g); *M*_T_ was N mass in litterbags of the last sampling (g) [10].

Differences in litter N release rate of different winter snowpack and species from the field were analyzed with One-way ANOVA (correcting the *P*-value using Bonferroni correction). The correlation coefficient (*r*) between N release rate and litter qualities, microbial biomass and environment temperature were analyzed with the Pearson correlation coefficient method. All statistical analyses were performed using the program SPSS 20.0 for Windows (SPSS Inc., Chicago, IL, USA).

## Results

### N release rate

**Figure 4:**
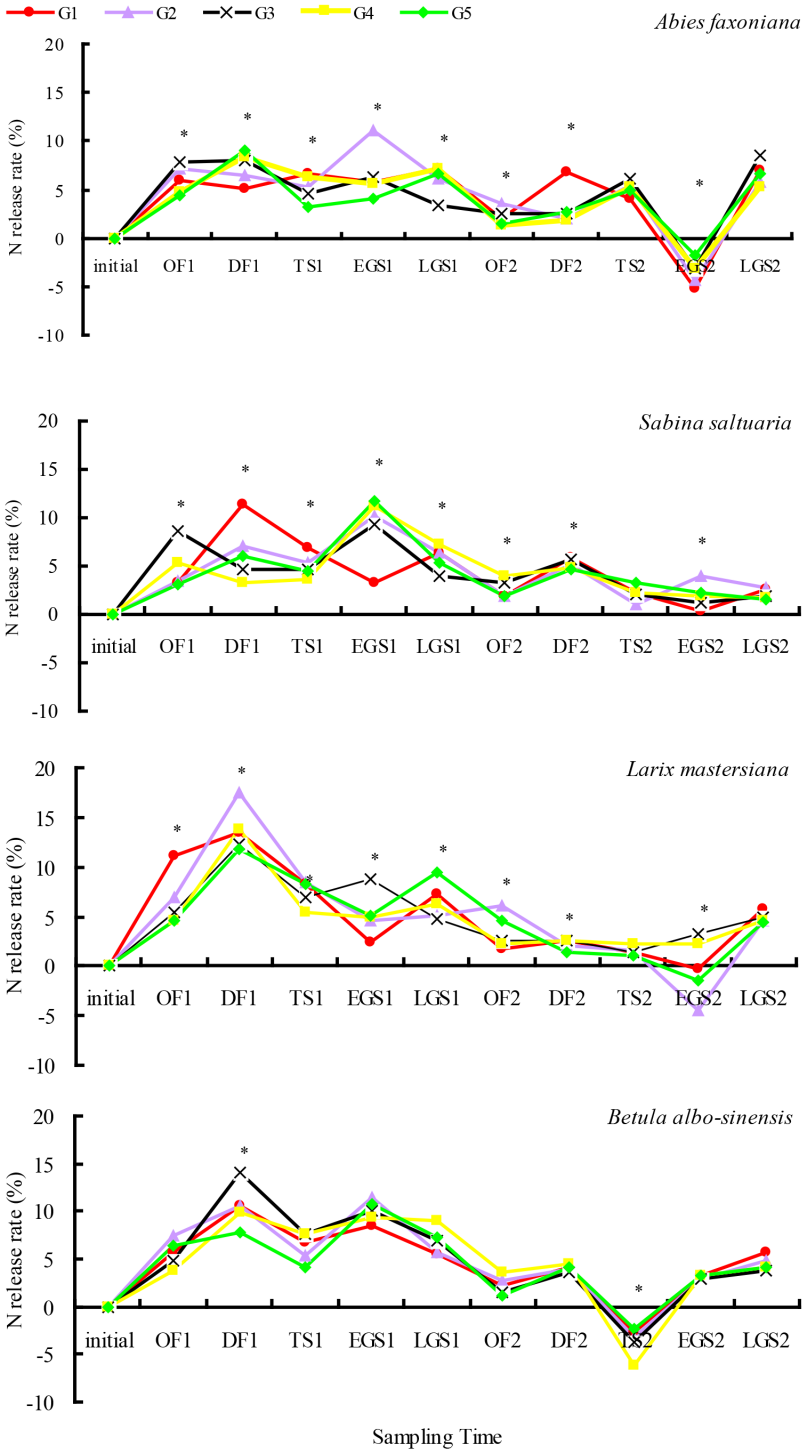
N release rate during litter decomposition from gap center to closed canopy at different decomposition stages over 2 years (mean ± *SD*, *n* = 5). The symbols “*” indicate the significant difference among treatments (LSD's multiple range test; *P*<0.05). Abbreviations: OF = onset of freezing stage; DF = deep freezing stage; TS = thawing stage; EGS = early growing season; LGS = late growing season; G1 = gap 1, gap center south; G2 = gap 2, gap center north; G3 = gap 3, canopy edge; G4 = gap 4, expanded edge; G5 = gap 5, closed canopy.

Over 2-year decomposition, litter N release rates were 41–49% for fir, 49–54% for larch, 44–47% for cypress and 47–52% for birch (Table 2). Cypress, fir and larch litter showed obvious N enrichment in the 2^nd^ growing season, whereas birch litter showed N enrichment in the 2^nd^ winter (Figure 4). Litter N release rates in both the first year and entire two-year decomposition were relative higher in gap center than in closed canopy regardless of species. However, litter N release rates during the first winter decreased along the snowpack gradient from gap centers to under closed canopy, regardless of species, but increased along this gradient during the first growing season. Compared with broadleaf litter, needle litter N release showed much more obvious changes from gap centers to under closed canopy. The statistical results showed that except in OF1, species significantly affected litter N release rate across the different critical periods throughout the whole 2-year study period; however snowpack significantly affected litter N release rate in OF1, DF1, OF2, DF2 and TS2 (Table 3).

**Table 2:** N release rate of four kinds of litter from gap center to closed canopy at different decomposition stages over 2 years (%).

Species	Site location	1st winter	1st growing season	1st year	2nd winter	2nd growing season	2nd year	Two years
*Abies faxoniana*	G1	20 a	13 b	33 b	13 a	2 b	15 ab	35 b
	G2	19 b	17 a	36 a	11 b	2 b	13 b	38 a
	G3	20 a	10 c	30 c	11 b	5 a	16 a	35 b
	G4	19 b	13 b	32 b	8 c	2 a	10 b	34 bc
	G5	17 c	11 c	27 c	9 c	5 a	14 ab	32 c
*Larix mastersiana*	G1	33 a	10 c	42 a	6 b	5 c	11 b	48 b
	G2	33 a	10 c	43 a	10 a	1 e	10 b	43 ab
	G3	25 b	13 a	38 b	7 b	8 a	15 a	46 a
	G4	24 bc	11.13 b	35 c	7 b	7 b	14 a	42 b
	G5	23 c	14 a	37 bc	7 b	3 d	10 b	40 b
*Sabina saltuaria*	G1	22 a	10 d	32 a	10 a	3 b	13 a	34 b
	G2	16 c	17 b	32 a	8 b	7 a	15 a	39 a
	G3	18 b	13. c	31 a	11 a	3 b	14 a	34 b
	G4	12 d	18 ab	31 a	11 a	4 b	15 a	34 b
	G5	11 d	19 a	30 a	10 a	4 b	14 a	34 b
*Betula albo-sinensis*	G1	24 b	14 a	37 a	3 a	9 a	12 a	46 b
	G2	23 b	17 a	41 a	3 a	8 a	11 a	48 ab
	G3	26 a	17 a	43 a	1 a	7 a	8 a	50 a
	G4	21 b	18 a	40 a	2 a	7 a	9 a	47 ab
	G5	18 c	18 a	36 a	3 a	7 a	10 a	44 bc

The same letter in the same column indicates no significant difference at 0.05 level.

*n* = 5.

G1, gap 1 (gap center south); G2, gap 2 (gap center north); G3, gap 3 (canopy edge); G4, gap 4 (expanded edge); G5, gap 5 (closed canopy).

**Table 3:** Effects of species (S) and winter snowpack (C) on N release rate in the alpine forest.

	OF1		DF1		TS1		EGS1		LGS1		OF2		DF2		TS2		EGS2		LGS2	
	df	*F*	df	*F*	df	*F*	df	*F*	df	*F*	df	*F*	df	*F*	df	*F*	df	*F*	df	*F*
*p*_S_	3	1.43	3	17.40**	3	16.32**	3	8.60**	3	17.58**	3	17.47**	3	15.18**	3	5.66**	3	23.58**	3	21.26**
*p*_C_	4	2.24**	4	1.96**	4	1.70	4	1.47	4	1.09	4	2.38**	4	1.73*	4	2.89*	4	1.04	4	1.13

OF, onset of freezing stage; DF, deep freezing stage; TS, thawing stage; EGS, early growing season; LGS, late growing season.

*p*_S_,effect of species; *p*_C_,effect of winter snowpack.

*indicates significant difference at *p*<0.05, ** indicates significant difference at *P*<0.01.

### Concentrations of litter MBC and MBN

**Figure 5:**
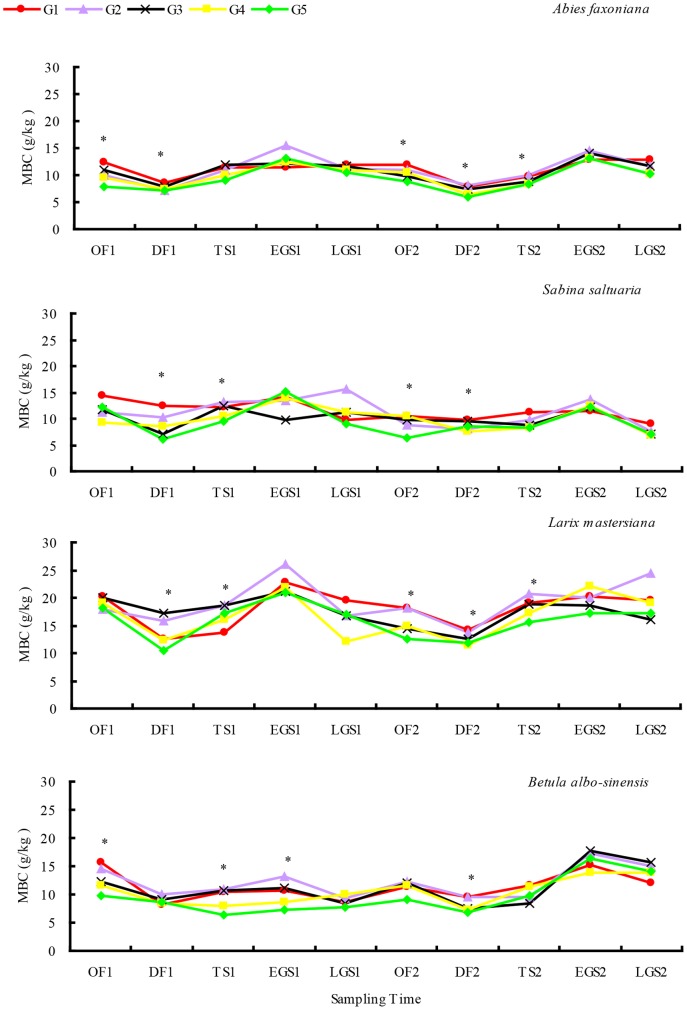
MBC during litter decomposition from gap center to closed canopy at different decomposition stages over 2 years (mean ± *SD*, *n* = 5). The symbols “*” indicate the significant difference among treatments (LSD's multiple range test; *P*<0.05). Abbreviations: MBC  = microbial biomass carbon; OF = onset of freezing stage; DF = deep freezing stage; TS = thawing stage; EGS = early growing season; LGS = late growing season; G1 = gap 1, gap center south; G2 = gap 2, gap center north; G3 = gap 3, canopy edge; G4 = gap 4, expanded edge; G5 = gap 5, closed canopy.

**Figure 6:**
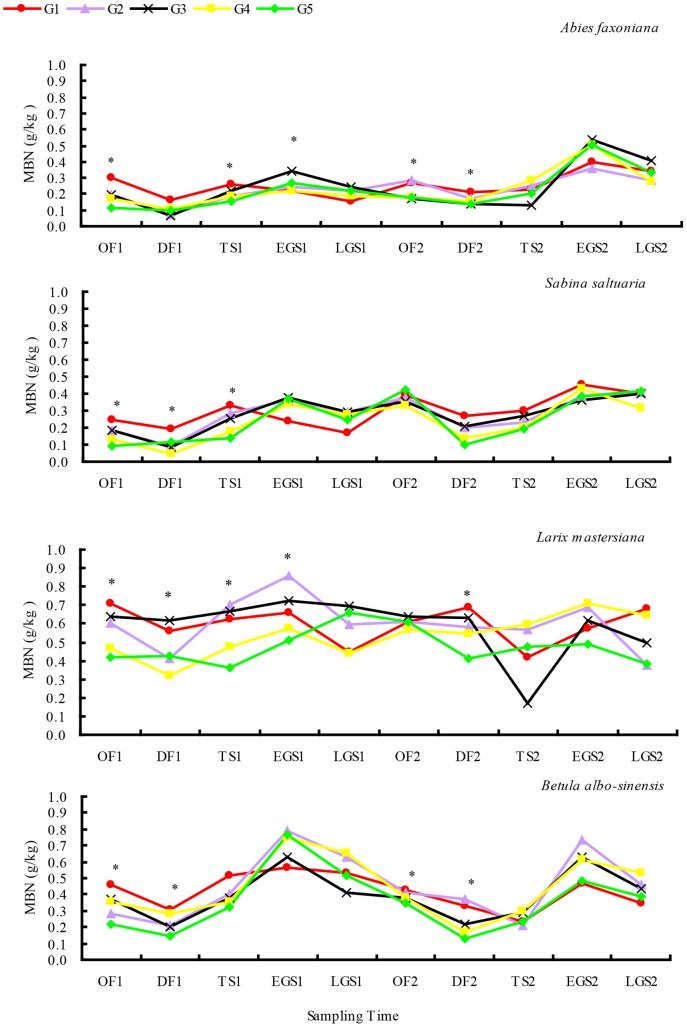
MBN during litter decomposition from gap center to closed canopy at different decomposition stages over 2 years (mean ± *SD*, *n* = 5). The symbols “*” indicate the significant difference among treatments (LSD's multiple range test; *P*<0.05). Abbreviations: MBN  = microbial biomass nitrogen; OF = onset of freezing stage; DF = deep freezing stage; TS = thawing stage; EGS = early growing season; LGS = late growing season; G1 = gap 1, gap center south; G2 = gap 2, gap center north; G3 = gap 3, canopy edge; G4 = gap 4, expanded edge; G5 = gap 5, closed canopy.

During 2-year decomposition, all four kinds of litter exhibited similar dynamics patterns of both MBC and MBN (Figures 5 and 6). Both MBC and MBN were higher in gap centers than under closed canopy during most of the decomposition stages. The highest MBC was observed at EGS1 and EGS 2, and the lowest at DF1 and DF2. MBN was also higher at EGS1 and lower at DF1 in the first decomposition year. Although the lower MBN was detected at both DF2 and TS2 in the second decomposition year, MBN at EGS2 showed the highest values in all two-year decomposition stages.

### Relative proportion of N release rates at each stage

**Figure 7:**
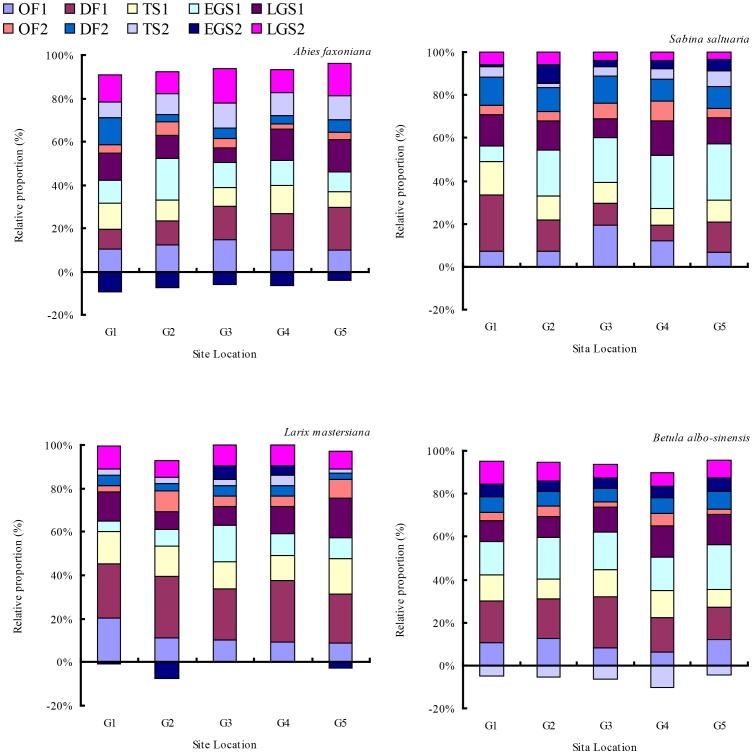
Relative proportion (%) at each decomposition stage to 2 years N release in different gaps. Abbreviations: OF = onset of freezing stage; DF = deep freezing stage; TS = thawing stage; EGS = early growing season; LGS = late growing season; G1 = gap 1, gap center south; G2 = gap 2, gap center north; G3 = gap 3, canopy edge; G4 = gap 4, expanded edge; G5 = gap 5, closed canopy.

The litter N release was mostly highest in the gap centers and lowest under closed canopy during the first year of decomposition irrespective of species (Figure 7). The relative proportions of N release over both winters in comparison to the entire two-year release increased with the increase of snow depth from gap center to closed canopy. Compared with closed canopy samples, gap center south samples were significantly higher in regards to the relative proportions of N release during both winters by 2% for fir, 12% for larch, 31% for cypress and 10% for birch. In contrast, the relative proportions of N release during both two growing seasons in comparison to the entire two-year release decreased with form gap center samples to samples from under closed canopy.

### Correlations

Amongst the factors studied, the frequency of soil freeze-thaw cycles was significantly related to the N release rate during decomposition stages in winter (Table 4). The N release rate was closely related to the soil accumulated temperature at OF1 and LGS1. MBC and MBN were significantly related to the N release rate at DF1 and OF2. The N release rates at TS, EGS and LGS in both decomposition years were closely related to the initial C concentration. The initial N concentration was closely related only to N release rate at EGS1 and TS2, but the initial P concentration was closely related to the N release rates at DF1, EGS1, DF2, OF2 and TS2. In addition, the N release rates at EGS1, OF2 and TS2 had significant correlation with C/N, C/P, and N/P ratios.

**Table 4:** Correlation coefficient (*r*) between decomposing litter quality, microbial biomass and environment temperature with N release rate.

	Sampling Time	C Concentration	N Concentration	P Concentration	Cellulose Concentration	Lignin Concentration	C/N	C/P	N/P
Nitrogen Release Rate	OF1	−0.08	−0.08	0.14	−0.35**	0.11	0.09	−0.08	−0.06
	DF1	−0.20	−0.09	044**	−0.32*	0.53**	0.18	−0.28*	−0.13
	TS1	−0.27*	−0.09	0.23	−0.35**	0.27*	0.14	−0.14	−0.08
	EGS1	−0.23*	0.31*	−0.43**	0.18	0.03	−0.32*	0.43**	0.39**
	LGS1	0.31*	0.16	0.03	0.41**	0.19	−0.14	−0.01	0.07
	OF2	−0.09	−0.24	0.32*	−0.17	0.05	0.26*	−0.30*	−0.26*
	DF2	−0.22	0.18	−0.38**	0.40	−0.24	−0.24	0.312*	0.24
	TS2	0.48**	−0.62**	0.29*	−0.44**	−0.73**	0.55**	−0.49**	−0.63**
	EGS2	−0.35**	0.31*	−0.15	0.51**	0.30*	−0.28*	0.24	0.31*
	LGS2	0.69**	0.14	0.05	0.06	0.21	−0.14	−0.02	−0.03
	Sampling Time	L/N	MBC	MBN	Frequency of Soil Freeze-thaw Cycle	Soil Accumulated Temperature	Surface Soil Average Temperature	Accumulated Positive Temperature	Accumulated Negative Temperature
Nitrogen Release Rate	OF1	0.22	0.20	0.24	0.28**	0.26*	0.23	0.10	0.29*
	DF1	0.70**	0.58**	0.63**	0.04	0.16	0.16	−0.02	0.10
	TS1	0.40**	0.24	0.46	0.25*	0.22	0.22	−0.20	0.15
	EGS1	−0.31*	−0.31	0.12	−0.07	−0.19	−0.18	−0.19	-
	LGS1	0.05	0.04	0.06	0.17	−0.30*	−0.19	−0.26*	−0.17
	OF2	0.31	0.29**	0.25*	0.17	−0.05	−0.13	−0.10	−0.12
	DF2	−0.47**	−0.18	−0.33	0.31**	0.22	0.22	0.05	0.23
	TS2	−0.16	−0.07	−0.04	0.21*	−0.01	−0.01	−0.02	0.01
	EGS2	−0.01	0.140	0.14	−	−0.11	−0.13	−0.12	−
	LGS2	0.10	0.277*	−0.05	0.15	0.18	0.18	0.15	0.01

OF, onset of freezing stage; DF, deep freezing stage; TS, thawing stage; EGS, early growing season; LGS, late growing season;

* indicates significant difference at *P*<0.05; ** indicates significant difference at *P*<0.01.

*n* = 60.

## Discussion

The hypothesis that N release rates from foliar litter will decrease along the snowpack gradient from gap centers to under canopy cover in winter was tested from the present results during 2 years' decomposition. The process of litter elements release in winter would be delayed by a decrease in winter snowpack, and this would then seriously affect the material cycling of the ecosystem [5]. In the present study, the relative proportions of N release in both winters compared to the entire two-year release increased with the increase of snow depth from gap center to closed canopy, but vice versa in growing season. It was also observed that the N release dynamic of needle litter and broadleaf litter had different responses to winter snowpack, which agree with the previous results [18], [32]. Moreover, the long-term relationship between N release and winter snowpack could mean that glacier ablation and snow melting under the effect of climate change would slow down the rate of N release from forest litter in this area, and thus affect ecosystem function and the main ecological processes.

In the process of decomposition over two years, the N release rates in different gaps were 41–49% for fir, 49–54% for larch, 44–47% for cypress, and 47–52% for birch. In different gaps, N release during the first year for each of the four types of litter accounted for more than 60% of the total during the two years. Thus the decomposition process over the 2 years could be divided into a rapid decomposition stage and a slow decomposition stage [33]. Moreover, the relative proportion to N release also indicated that the first year of litter decomposition was the main stage for N release. In this stage the carbohydrate, N and P all underwent rapid leaching and degradation process. This resulted in this stage being the main stage for mass loss [13] and element release [34]. During the second year of decomposition, increasing refractory material slowed down the decomposition and nutrient release [35], [36]. The results of this research support all of the above conclusions. However, the mode of N release during the research (enrichment-release-enrichment) showed results that conflicted with existing research [16], [37], [38]. It illustrates that the seasonal snowpack in the alpine forest affects the mode of litter N release.

The initial qualities of litter, environmental temperature, litter microbial biomass and soil freeze-thaw cycles were the main contributing factors to litter decomposition rates. In existing research, N concentration, phosphorus concentration, lignin concentration, and C/N, C/P and L/N ratios were common indexes for litter quality. In addition C/N and L/N ratios could be used as indicators of the decomposition rate in most instances [16], [39]. Hornsby et al. [40] found that decomposition rate would increase with rising temperature. The result of Singh et al. [41] further proved that temperature had a significant effect on decomposition. In this research, C concentration, P concentration, C/N, and C/P ratios and N release rate maintained a high correlation. This is in accord with a study by Gosz et al. [42]. The litter C/N and C/P ratios were important factors to N release and enrichment. In the freeze-thaw cycles frequent during this study (from October 26, 2010 to April 19, 2011 and from November 27, 2011 to April 28, 2012), the soil temperature showed the ranked order of G1>G2>G3>G4>G5. The temperature fluctuations increased gradually under different winter snowpack conditions. The day-time temperature fluctuated slower than air temperature, and the temperature at night was significantly higher than that of air temperature. During the growing season (from April 19, 2011 to November 8, 2011 and from April 28, 2012 to October 29, 2012), topography, gap size and gap position, and other heterogeneity factors resulted in different degrees of direct sunlight on the experimental plots' surfaces. In different gaps, the soil temperature conditions showed large fluctuations, and the amplitude increased with the gap to forest decline (Figure 2). During the snow cover stage, the correlation coefficient between N release rate, surface soil average temperature, and soil accumulated positive and negative temperature proved the hypothesis that: winter snowpack could affect the process of litter decomposition through changing the environmental temperature. This is in accord with the study of Tan et al. [21], but contrary to the finding of Parton et al. [1]. What's more, because of the isolation effect of winter snowpack, the microbial groups were more abundant in snow-covered sites than in sites not under winter snowpack [43]. Concentrations of leaf litter MBC and MBN were also relatively higher, even without snowpack in winter (Figures 5 and 6), which can promote the process of N release.

The statistical results showed that species had a significant affect on litter N release rate across the different critical stages of 2 years, so the quantity of litter was a central factor in litter decomposition [44]. The effect of different winter snowpack on N release rates was also key. The winter snowpack significantly affected N release rate in OF (two years), DF1 (two years) and TS2. This illustrated how snowpack in winter and snow melting in TS could affect N release significantly. Because of the difference in initial N concentration, the qualities of different types of litter could affect the N release rates during the 2 years' decomposition. Regarding litter N release dynamics during the 2 years, N release rates in winter were always greater in G1, G2, G3 and G4 than those in G5, indicating that winter snowpack significantly improved N release: The biggest difference was visible between G1 and G5. During the growing season, the relative proportion to N release rate in G5 was significantly greater than in G1, G2, G3 and G4. Therefore, winter snowpack significantly improved N release proportionally to the four litter types during winter. Under snowpack in winter, the relative proportion to N release of each of the four types of litter were elevated by about 7% for fir, 6% for larch, 7% for cypress and 5% for birch. These results indicated that the difference between freezing, melting and freeze-thaw cycles caused by diversity in microenvironments under different winter snowpack had a significant influence on litter N release. Additionally, obvious enrichment demonstrated that decomposer competition for N in late decomposition substantiated that in these alpine forest ecosystems, N availability is a limiting factor in ecosystem processes.

Because of the effect of the winter snowpack, different seasonal stages had different effects on litter decomposition, and thus N release in different stages showed different responses. Between winter snowpack (G1, G2, G3 and G4) and no winter snowpack (G5) the dynamic of N release were significantly different, so different depths of snow could also significantly influence the process of N release. This may be because the winter snowpack (G1, G2, G3 and G4), provided conditions where ambient temperature was relatively stable, with a relatively suitable environment for the activity of decomposers [24], [45], [46]. Additionally, during the melting stage, the strong leaching under winter snowpack areas also promoted the process of N release [47], [48].

## Conclusions

The rate of N released from foliar litter N in gap centers was comparable with under closed canopies over the two-year decomposition study in this alpine forest. However, N release rates from foliar litter decreased along the snowpack gradient from gap centers to under the closed canopy in winter, while the opposite occurs during the growing season. Compared with broadleaf litter, needle litter N release showed much stronger responses to the changes of snow cover in winter and sunshine in the growing season (as produced by either closed canopy cover or being in the center of a gap). The results suggested that forest gaps plays an important role in litter N release, and a decrease in winter snowpack might slow down litter N release in the alpine forest in the scenario of winter warming.
